# Dentists' Perception and Clinical Use of Preformed Metal Crowns to Restore Primary Molar Defects in Chengdu City, China: A Cross-Sectional Study

**DOI:** 10.1155/2021/6200083

**Published:** 2021-08-02

**Authors:** Qingsong Jiang, Xinyi Zeng, Yan Wang, Jing Zou, Qiong Zhang

**Affiliations:** State Key Laboratory of Oral Diseases, National Clinical Research Center for Oral Diseases & Department of Pediatric Dentistry, West China Hospital of Stomatology, Sichuan University, Chengdu, China

## Abstract

**Background:**

Preformed metal crowns (PMCs), as an effective technique recommended for the enduring restoration of primary molar defects, have not been widely implemented in China as well as that in Western countries. This study is aimed at assessing the knowledge on the clinical use of the PMC technique and its effective factors among dentists in Chengdu, China. Thus, the present study is aimed at providing the best available evidence on clinical decision-making to manage dental caries in children and the overall benefits.

**Methods:**

The self-designed questionnaire in this study consisted of two main sections, including the sociodemographic characteristics of the respondents and their perception and use of PMCs. The anonymous questionnaire was distributed among 1000 dentists practicing in Chengdu via SO JUMP.

**Results:**

The response rate was 45%. Most respondents (69.6%) did not use PMCs. Academic qualification, working specialty, and professional experience were associated with dentists' use of PMCs. The lack of knowledge about the PMC technique was the main obstacle to the dental practitioners' use of PMCs (41.7%). The lack of understanding of the rationale behind PMCs was the main reason for parents' acceptance of PMC restorations (43.6%). Attending continuing education programs was the main approach to learning the PMC restorative technique (59.1%).

**Conclusions:**

The clinical use of PMCs was not very popular in Chengdu city. To promote this restorative technique, knowledge and practical training should be incorporated into routine courses of undergraduate dental education.

## 1. Introduction

Dental caries, a worldwide oral health burden, affects 60–90% of children and an overwhelming majority of adults in most industrialized countries [1]. Early childhood caries (ECC) is an aggressive form of dental caries in preschoolers, which, left untreated, can give rise to the rapid development of extensive lesions in primary teeth, causing pain, complicated systemic infections, and financial burden. According to the report of the Fourth National Oral Health Epidemiological Survey in mainland China, the prevalence of ECC in children aged 3, 4, and 5 years was 50.8%, 63.6%, and 71.9%, respectively. However, the constituent rate of filled teeth was only 1.5%, 2.9%, and 4.1%, respectively [2].

Maintenance of the primary dentition in a nonpathological condition is vital for oral health, craniofacial development, and the overall wellbeing of children. Therapeutic strategies of the ECC are different from those of the permanent dentition because of the specific characteristics of the anatomical morphology and histological structures of primary teeth and even the children's psychological behavior [3]. Therefore, the treatment of extensively damaged primary teeth poses a challenge for pediatric dentists as three critical considerations have to be kept in mind: children's behavioral management, preservation of the tooth structure, and parental satisfaction. In contemporary dentistry, the defects of primary molars are mainly treated with restorative treatments, which include direct filling and full-coverage restorations [4].

Preformed metal crowns (PMCs), also known as stainless steel crowns (SSCs), are recommended for enduring restorations of primary molars and the temporary restoration of newly erupted first permanent molars on different occasions, including severely decayed or fractured teeth, hypoplastic or hypomineralized teeth, primary molars after pulpotomy or pulpectomy procedures, teeth with extensive wear, abutments for space maintainers, and children with a high risk of caries treated under local anesthesia [5]. Compared with conventional therapeutic materials, PMCs prevent caries by covering the entire dental crown surfaces and isolating the tooth structure from the oral microorganisms. Also, they exhibit greater longevity and fewer retreatment needs; thus, they constitute a preferred option for restoring primary molars [5–7]. PMC restoration is widely accepted by dentists, children, and their parents in some developed countries, such as the UK and New Zealand [8, 9]. However, to the best of our knowledge, PMCs are not widely accepted as an ideal treatment option in China, and dentists' perception, clinical use, and knowledge of PMCs are considered inadequate.

Chengdu, located at the western edge of the Sichuan Basin, is the provincial capital of Sichuan Province. According to a cross-sectional study in Sichuan Province in 2016, the prevalence of dental caries in children aged 3–5 years was 63.55%, and the mean DMFT (decayed, missing, and filled teeth) was 3.28. The significant caries index reached 8.05, and this value was higher in urban areas than in rural areas. Relative to the high rate of decayed teeth, the prevalence of restored teeth was only 0.97% in this group [10]. The prevalence of caries and health risks due to dental caries in children and adolescents in Sichuan is an essential factor in governmental investment and planning for dental services and public health practitioners' and dentists' delivery and practice of dental services.

Dentists played an important role in the establishment of early childhood oral health and overall health management. Therefore, this study conducted a questionnaire survey on dentists in Chengdu city to assess the clinical use of PMCs and factors affecting it from dentists' perspective to promote the clinical application of PMCs. By evaluating dentists' cognition, popularization, and implementation level of PMC, we can formulate preventive and therapeutic strategies for children's oral diseases and provide a scientific basis for strengthening medical care measures.

## 2. Methods

### 2.1. Study Design

The present study was conducted on the general dental practitioners, pediatric dental specialists, and other general dentists practicing in Chengdu, China, who are members of the Sichuan Society of Stomatology. The relevant questionnaire was presented to the participants via SO JUMP (https://www.wjx.cn/), an online questionnaire platform. The survey was anonymous, and therefore, no consent was required. A covering letter listing the purposes of this study was sent along with the questionnaire.

### 2.2. Questionnaire Development

The questionnaire (21 questions) consisted of two main sections, including multiple-choice questions and questions with blank spaces. One section dealt with the sociodemographic characteristics of the participants, including gender, age, academic qualification, working specialty, professional qualification, years of practice, nature of work units, and the number of children treated per week. The other section consisted of questions on their use and perception of PMCs, including months of applying PMCs, the charge of PMC restorations, the number of PMCs delivered per week, the age of young patients treated with PMCs, approaches to learning this technique, indications of using PMCs, and the reasons for dentists and young patients to reject PMCs (attached as an appendix).

Pilot testing was conducted on 10 dentists contacted by WeChat, a mobile text and voice messaging communication service developed by Tencent in China, to make sure it was comprehensible and acceptable to the participants. Neither the questions nor the answers were modified after the pilot study.

### 2.3. Data Analysis

The data from the questionnaire were analyzed with SPSS (Version 24, SPSS Inc., Chicago, IL, USA). Descriptive statistics were used to analyze respondents' demographic characteristics and approaches used by dentists to learn the PMC restorative technique. Logistic regression analysis was used to reveal the association between demographic characteristics and the use of PMCs. Multiple responses were used to analyze the reasons why dentists and patients rejected PMCs and dentists' reported indications to use PMCs. The multivariate logistic regression model was performed with the variables with a univariate test at *P* < 0.05. A *t*-test was applied to compare the mean number of children treated and the number of PMCs used by general and pediatric dentists per week, respectively. The level of significance was defined at *P* < 0.05.

## 3. Results

The questionnaire return ratio was 45%, and 450 complete samples were obtained.  Table 1 shows the demographic characteristics of the studied dentists and the association with the use of PMCs; 60.2% of the respondents were female dentists, with 49.3% of them 30–39 years old, and 66.9% had bachelor's degrees. The majority (80.2%) of the respondents were general dental practitioners. More than half of the dentists (54.9%) were resident doctors; 183 (40.7%) had worked for 10–19 years, and almost half of them (52.4%) worked in private dental clinics/hospitals.

According to the survey, most dentists (69.6%) in Chengdu city did not use PMCs to restore primary molar defects. Logistic regression analysis was used to analyze the association between dentists' demographic characteristics and the use of PMCs. The multivariate logistic regression model showed that a junior college degree and lower degrees, being a general dental practitioner, and longer working years were negatively associated with the dentists' use of PMCs.

As showed in Table 2, dentists' reluctance to use PMCs was due to a lack of knowledge to use PMCs (41.7%), low charges/low input-output ratio (14.9%), other technical limitations (e.g., local anesthesia) (9.3%), a lack of compliance by children (5.6%), aesthetic concerns (5.4%), unawareness of PMCs (4.2%), and other reasons (18.9%). Parents rejected PMC restorations because of a lack of knowledge of the merits (43.6%), the price (24.2%), aesthetic concerns (22.0%), and children's noncompliance (10.2%).

In this study, 30 pediatric dentists and 100 general dental practitioners used the PMC restorative technique. The mean number of children treated by pediatric dentists per week (63.70) was almost three times that by general dental practitioners (20.56) (*P* < 0.001). Accordingly, the mean number of PMCs used by pediatric dentists per week (7.73) was nearly three times that used by general dental practitioners (2.92) (*P* < 0.05) (Table 3).

This questionnaire also showed that most children who received PMC restorations were 3–6 years of age (57.1%). However, children < 3 years of age (8.8%) seldom received PMC restorations. Moreover, 59.1% of the respondents gained the PMC restorative technique skills through attending continuing education programs. Besides, 29.9% learned it by themselves, and only 10.9% were educated in college.

We also investigated the dentists' perception of indications for the PMC restorative technique. Almost all dental practitioners believed that PMCs should be used to restore primary molars with multisurface caries (94.6%) and after endodontic treatment (86.9%). More indications that were stated for the implementation of the PMC restorative technique are listed in Table 4.

## 4. Discussion

ECC has become a worldwide disease, affecting both the oral and general health of children. PMCs have been demonstrated to be the most reliable and durable restorations for multiple lesions in primary molars, particularly for children at high risk for caries [11–13]. The International Caries Consensus Collaboration (ICCC) recommended that the Hall technique be selected for primary teeth with shallow/moderate and deep caries [14, 15]. The success rates of placing PMCs using the conventional method or Hall technique were 94% and 97%, respectively [16]. Another retrospective study also showed that at the first follow-up visit (after a mean of 9.9 months), the clinical and radiographic success rates of PMC restoration for primary molars were 98.9% and 97.7%, respectively. At the second follow-up visit (after a mean of 20.1 months), the clinical and radiographic success rates were 97.4% and 94.9%, respectively [17]. A study from Hong Kong showed that PMCs were more frequently employed in patients with special needs (patients requiring medical management, health care intervention, and/or use of specialized services or programs) under 12 years of age [18]. However, the PMC restorative technique is not popular in the Chinese mainland. This study investigated the use and views of the PMC technique among general dental practitioners and pediatric dentists in Chengdu, China. The present study is the first to investigate dentists' perception and clinical use of PMCs in the Chinese mainland. The study findings contributed to the knowledge of the use of PMC and the limiting factors.

The results showed that approximately one-third of the respondents (30.4%) used PMCs to restore the defects in primary molars in their clinical practice, consistent with the results of previous studies in Germany and the UK [19, 20]. In the study conducted in Germany, general and pediatric dentists were investigated, and only 34% of the respondents routinely used the PMC technique. Additionally, in the study conducted in the UK, 3% of the participants routinely used PMCs, with 15% infrequently and 82% never using it. Compared with the study conducted in the UK, a limitation of the present study was the lack of frequency classification (routinely, infrequently, and never). However, the superiority was that the sample size (450) in the present study was larger than that in the studies in Germany (104) and the UK (93).

According to the present study, the main deterrent to the use of PMCs was a lack of knowledge of dental practitioners to place PMCs (41.7%). However, a previous study showed that only 11.7% of dentists in Germany thought the lack of knowledge to place the crowns prevented them from using PMCs [19]. These findings highlighted the importance of learning how to place PMC restorations. The results also showed that the application of PMCs was associated with the academic qualification, working specialty, and working experience of dentists. Dental practitioners with higher academic qualifications were more likely to implement the PMC restorative technique, which might be attributed to the training of the PMC technique in different educational stages. Besides, pediatric dental specialists treated more children and used more PMCs than general dental practitioners, consistent with a recent study [21]. In that study, the authors found that pediatric dentists are more likely to place PMCs compared to general dental practitioners (OR = 3.2, *P* < 0.001). They hypothesized that the difference resulted from insufficient training in PMCs at the dental undergraduate level and continuing education courses for general dental practitioners. In addition to the professional training, we believe that the difference also resulted from the fact that pediatric dental specialists have a higher professional understanding of children's oral problems and treat more young patients. Additionally, dentists with less working experience were more likely to use PMCs, which might be due to a higher success rate of PMC restorations and ever-increasing recommendations by experts in recent years [13, 22]. Therefore, to promote the implementation of this technique, the importance and the knowledge on the PMCs technique should be imparted during undergraduate education, encouraging dental undergraduates to learn and practice it systematically when they participate in postgraduate pediatric dentistry courses. For those who have not participated in such training, especially the general dental practitioners, continuing education programs could be a preferred approach. Santamaria et al. [19] showed that the use of PMCs was taught as a restorative procedure in 96% of German dental schools, but 27% of dental schools did not provide this practical training. The training of the PMCs restorative technique in Chinese dental schools, however, is not clear. Future studies could investigate the use and teaching of the PMCs technique in dental schools in different regions in China.

The perceived technique complexity also appeared to be associated with practitioners' reluctance to use PMCs, with 28.9% of the respondents believing that it was one of the main obstacles to implementing the PMC technique in a previous study [19]. Perhaps some of the investigated dentists in the present study had no idea about the operational process of PMC restoration, which might contribute to the low constituent ratio of technique complexity. The low economic benefit was reported as the second leading limitation to the use of PMCs in the present study, with 14.9% of respondents believing that it was a reason for their reluctance to apply this technique. This issue was also analyzed in a previous study, which investigated general dental practitioners' views on using PMCs [20]. In that study, some general dental practitioners believed that they would develop the use of PMCs in their daily practice if the charge could increase. On the other hand, the cost was also the second leading reason (24.2%) for patients' hesitation to choose a PMC restoration. Perhaps improving the operational proficiency or developing a new technique to simplify the operational process could increase the input/output ratio and decrease the expense of patients, which would promote the use of PMCs. In addition, children's noncompliance and the unaesthetic appearance of PMCs were other limitations for dentists to use them [19, 20, 23]. The Hall technique has been demonstrated to exhibit a high success rate (97%) over five years in the UK and is regarded as one of the several caries biological management options [16]. It can restore carious primary molar teeth by seating correctly sized PMCs over the tooth and sealing the carious lesion using the glass-ionomer cement. The Hall technique could be accepted by general dental practitioners quickly given there is no need for local anesthesia, tooth preparation, or removal of the carious tissue. Moreover, BaniHani et al. [24] reported that the Hall technique only costs about half the conventional approach. Therefore, general and pediatric dentists could apply the Hall technique to appropriate cases in their clinical practice, especially in young children. However, we believe that it also poses some challenges for the dentist to select the correct size of the PMC at first sight.

Apart from dentists' perception, the use of the PMC technique was also affected by the children's and their parents' views. The results showed that the main reason for patients' refusal was a lack of understanding of the merits of PMC restorations (43.6%). Therefore, dental practitioners should attach importance to explaining the rationale for the PMC restorative technique to the parents before treatment. Furthermore, the dentists' attitudes and effective communication are crucial to gaining the trust and understanding of children and their guardians [24]. Moreover, it is necessary to promote children's oral health knowledge and raise public awareness of the importance of childhood dental health.

Almost all (94.6%) dentists believed primary molars with multisurface caries needed PMCs, consistent with a previous study in Germany [19], where 88.6% of the respondents had the same idea. Ebrahimi et al. [25] showed that PMCs had the lowest failure rates after 12 months in terms of restoring primary molars with multisurface defects. Besides, PMCs are also indicated in the following situations: primary molars after pulpectomy or pulpotomy procedures, molars with developmental defects (e.g., dentinogenesis imperfecta, amelogenesis imperfecta, and enamel hypoplasia), tooth fracture, or teeth with extensive surface loss, high caries risk, infraocclusion, etc. [26]. In clinical practice, however, the indications, dentists' experience, appearance, cost, young patients' compliance, and contraindications should be considered for the choice of PMC restoration.

The current study showed that the majority (57.1%) of patients who received PMC treatment were 3–6 years of age, and only 8.8% of patients were<3 years of age. The ECC prevalence for children aged 1, 2, and 3 years in mainland China was 0.3%, 17.3%, and 40.2%, respectively [27]. PMC restoration has a high success rate in ECC treatment [22, 28]. However, the conventional method to place crowns is more complicated than the Hall technique, requiring a higher level of cooperation by young patients [16, 20], which might be an obstacle preventing children < 3 years of age from receiving PMC restorations. The Hall technique is considered more comfortable for children to tolerate [29]. Therefore, it should be incorporated into routine treatments, especially for young children, who cannot easily tolerate complicated treatments and local anesthesia. On such occasions, the dentist would be prompted to improve the ability to select the right size of PMCs immediately.

Overall, the present study showed that most dental practitioners did not implement the PMC technique in Chengdu, China. The main reasons included a lack of knowledge to use PMCs, low charge/low input-output ratio, other technical limitations (such as local anesthesia), and children's noncompliance. Therefore, knowledge and practice of using PMCs should be incorporated into the routine educational curriculum of dental students during their undergraduate studies to enhance their understanding of this technique and encourage them to implement it to treat primary molars in their future clinical practice. In addition, continuing education programs are required to provide general dental practitioners with the means to learn the PMC restorative technique. Moreover, the Hall technique can be considered a routine method to place PMCs, reducing the complexity of the practice and increasing children's cooperation.

There were two main limitations in the present study, one of which was the nonresponse bias. The response rate in the present study was only 45%, a little bit higher than that in other studies focusing on dentists [19, 20]. The low response rate might partly be because some dentists were unfamiliar with PMCs, which could lead to an overestimation of the rate of implementing PMC restorations. Another limitation was the selection bias. All the participants were members of the Sichuan Society of Stomatology, who were not the optimum representatives of the whole population of dentists in China.

Since dentists' lack of knowledge to use PMCs is one of the main obstacles to PMC restorations, therefore, further studies should focus on the views of dental school teachers and students on teaching the PMC technique.

## 5. Conclusions

Based on the results of this study, the PMC restorative technique was implemented at a low rate in Chengdu, China. The main obstacles include dentists' lack of knowledge to use PMCs appropriately and patients' lack of knowledge about their merits. Dental undergraduate curricula and continuing education programs are responsible for promoting the use of PMCs.

## Figures and Tables

**Table 1:** Demographic characteristics of the studied dentists and the association with the use of PMCs.

	*n* (%)	Univariate		Multivariate	
		OR (95% CI)	P	OR (95% CI)	P
Gender				—	
Male	179 (39.8)	0.716 (0.471~1.088)	0.117		
Female	271 (60.2)	1.000			
Age (year)				—	
<30	91 (20.2)	1.156 (0.376~3.554)	0.801		
30-39	222 (49.3)	1.300 (0.447~3.784)	0.630		
40-49	119 (26.5)	0.876 (0.288~2.663)	0.816		
≥50	18 (4.0)	1.000			
Academic qualification					
Junior college degree and below	71 (15.8)	0.149 (0.061~0.367)	<0.001^∗∗∗^	0.094 (0.033~0.267)	<0.001^∗∗∗^
Bachelor degree	301 (66.9)	0.648 (0.389~1.080)	0.096	0.674 (0.385~1.181)	0.168
Master degree and above	78 (17.3)	1.000		1.000	
Working specialty					
General dentist	361 (80.2)	0.307 (0.171~0.550)	<0.001^∗∗∗^	0.299 (0.158~0.565)	<0.001^∗∗∗^
Other dental specialist	35 (7.8)	0.200 (0.075~0.537)	0.001^∗∗^	0.434 (0.144~1.307)	0.138
Pediatric dental specialist	54 (12.0)	1.000		1.000	
Professional title				—	
Resident doctor	247 (54.9)	0.894 (0.393~2.032)	0.789		
Attending doctor	171 (38.0)	1.529 (0.666~3.507)	0.317		
Association senior doctor and above	32 (7.1)	1.000			
Working experiences (year)					
0-9	164 (36.4)	3.756 (2.026~6.964)	<0.001^∗∗∗^	3.807 (1.994~7.271)	<0.001^∗∗∗^
10-19	183 (40.7)	2.276 (1.224~4.234)	0.009^∗∗^	2.244 (1.174~4.289)	0.015^∗^
≥20	103 (22.9)	1.000		1.000	
Nature of working units					
Private dental clinic/hospital	236 (52.4)	0.566 (0.292~1.097)	0.092	1.242 (0.577~2.676)	0.579
Dental department in public general hospital	171 (38.0)	0.451 (0.226~0.901)	0.024^∗^	0.670 (0.313~1.436)	0.303
Public specialized dental hospital	43 (9.6)	1.000		1.000	

OR: odds ratio. 95% CI: 95% confidence interval. ^∗^*P* < 0.05; ^∗∗^*P* < 0.01; ^∗∗∗^*P* < 0.001.

**Table 2:** Reasons for rejecting the use of PMCs by dentists and parents.

		*n*	Percent of responses (%)	Percent of cases (%)
Reasons for rejecting the use of PMCs by dentists	Lack of knowledge to use PMCs	210	41.7	67.1
	Low charges/low input-output ratio	75	14.9	24.0
	Other technical limitations	47	9.3	15.0
	Noncompliance of children	28	5.6	8.9
	Aesthetic concerns	27	5.4	8.6
	Unawareness of PMCs	21	4.2	6.7
	Other reasons	95	18.9	30.4
	Total	503	100.0	160.7

Reasons for rejecting the use of PMCs by parents	Incomprehension of merits	103	43.6	82.4
	Prices	57	24.2	45.6
	Aesthetic concerns	52	22.0	41.6
	Noncompliance of children	24	10.2	19.2
	Total	236	100.0	188.8

PMCs: preformed metal crowns.

**Table 3:** The comparison between general and pediatric dentists in terms of the number of children treated and PMCs used per week.

	Mean			
	General dentists	Pediatric dentists	*t*	*P*
Number of children treated per week	20.56	63.70	5.615	<0.001^∗∗∗^
Number of PMCs used per week	2.92	7.73	2.736	0.010^∗^

PMCs: preformed metal crowns. ^∗^*P* < 0.05; ^∗∗∗^*P* < 0.001.

**Table 4:** Dentists' reported main indications to use PMCs for primary molar restoration.

Main indications of using PMCs	*n*	Percent of responses (%)	Percent of cases (%)
With multisurface caries	123	20.5	94.6
After endodontic treatment	113	18.8	86.9
Form anomalies (e.g., enamel aplasia)	97	16.2	74.6
Dental tissues with large defects and fractures	97	16.2	74.6
High carious risk	92	15.3	70.8
Infraocclusion	78	13.0	60
Total	600	100.0	461.5

PMCs: preformed metal crowns.

## Data Availability

All data generated and analyzed in this study are included within the article or available from the corresponding author on reasonable request.

## Abbreviations

PMCs: Preformed metal crowns
ECC: Early childhood caries
SSCs: Stainless steel crowns
DMFT: Decayed, missing, and filled teeth
OR: Odds ratio
95% CI: 95% confidence interval.

## Supplementary Materials

Supplementary Materials The questionnaire used in this study was attached as an appendix in the supplementary material.
