# Supplementary material for: Dentists' Perception and Clinical Use of Preformed Metal Crowns to Restore Primary Molar Defects in Chengdu City, China: A Cross-Sectional Study
Source: Biomed Res Int. 2021 Aug 2;2021:6200083. doi: 10.1155/2021/6200083 (PMC8355980; doi:10.1155/2021/6200083)
Supplement: Supplementary Materials — The questionnaire used in this study was attached as an appendix in the supplementary material. [file 6200083.f1.doc]

Questionnaire used in this study

1. Gender:

A. Male

B. Female

2. Age (year)

A. <30

B. 30-39

C. 40-49

D. ≥50

3. Academic qualification

A. Junior college degree and below

B. Bachelor degree

C. Master degree and above

4. Working specialty

A. General dentist

B. Pediatric dental specialist

C. Other dental specialist

5. Professional title

A. Resident doctor

B. Attending doctor

C. Association senior doctor and above

6. Working experiences (years)

7. The address of your working unit

8. Nature of working units

A. Private dental clinic/hospital

B. Dental department in public general hospital

C. Public specialized dental hospital

9. Does your working unit belong to teaching or non-teaching medical institutions?

A. teaching medical institutions

B. non-teaching medical institutions

10. How many children visit your clinics per week?

11. Do you use PMCs in your daily practice?

A. Yes

B. No

12. Why don’t you use of PMCs in your clinics ? (multiple selection question)

A. Unawareness of PMCs

B. Low charges / low input-output ratio

C. Aesthetic concerns

D. Non-compliance of children

E. Lack of knowledge to use PMCs

F. Other technical limitations

G. Other reasons

13. How long have you applied PMCs in your daily practice? (years)

14. The indications do you think PMCs should be used? (multiple selection question)

A. Teeth after endodontic treatment

B. Teeth with multi-surfaces caries

C. Teeth with form anomalies (e.g. enamel aplasia)

D. Dental tissues with large defects and fractures

E. Infra-occlusion

F. Children with high carious risk

15. The charge of PMCs restoration in your working unit.

RMB

16. How many PMCs do you applied per week?

17. The age of your patients receiving PMC restoration

A. <3

B. 3-6

C. >6

18. Have your patients ever rejected the use of PMCs?

A. Yes

B. No

19. Why do the parents of your patients reject the use of PMCs(multiple selection question)

A. Prices

B. Aesthetic concerns

C. Incomprehension of merits

D. Non-compliance of children

20. Where did you lean this technique?

A. I learned it by myself

B. I learned it in college

C. I learned it in continuing education program

21. Would you like to improve your PMCs restoration skill ?

A. Yes

B. No
